# MoMyb1 is required for asexual development and tissue-specific infection in the rice blast fungus *Magnaporthe oryzae*

**DOI:** 10.1186/s12866-015-0375-y

**Published:** 2015-02-19

**Authors:** Yanhan Dong, Qian Zhao, Xinyu Liu, Xiaofang Zhang, Zhongqiang Qi, Haifeng Zhang, Xiaobo Zheng, Zhengguang Zhang

**Affiliations:** Department of Plant Pathology, College of Plant Protection, Nanjing Agricultural University, and Key Laboratory of Integrated Management of Crop Diseases and Pests, Ministry of Education, Nanjing, 210095 China

**Keywords:** *Magnaporthe oryzae*, Conidiogenesis, Stress response, Cell wall integrity, Pathogenesis

## Abstract

**Background:**

The Myb super-family of proteins contain a group of functionally diverse transcriptional activators found in plant, animal and fungus. Myb proteins are involved in cell proliferation, differentiation and apoptosis, and have crucial roles in telomeres. The purpose of this study was to characterize the biological function of Myb1 protein in the rice blast fungus *Magnaporthe oryzae*.

**Results:**

We identified the *Saccharomyces cerevisiae BAS1* homolog *MYB1* in *M. oryzae*, named MoMyb1. MoMyb1 encodes a protein of 322 amino acids and has two SANT domains and is well conserved in various organisms*.* Targeted gene deletion of *MoMYB1* resulted in a significant reduction in vegetative growth and showed defects in conidiation and conidiophore development. Quantitative RT-PCR analysis revealed that the transcription levels of several conidiophore-related genes were apparently decreased in the Δ*Momyb1* mutant. Inoculation with mycelia mats displayed that the virulence of the Δ*Momyb1* mutant was not changed on rice leaves but was non-pathogenic on rice roots in comparison to the wild type Guy11. In addition, ∆*Momyb1* mutants showed increased resistance to osmotic stresses but more sensitive to cell wall stressor calcofluor white (CFW). Further analysis revealed that MoMyb1 has an important role in the cell wall biosynthesis pathway.

**Conclusion:**

This study provides the evidence that MoMyb1 is a key regulator involved in conidiogenesis, stress response, cell wall integrity and pathogenesis on rice roots in the filamentous phytopathogen *M. oryzae*.

**Electronic supplementary material:**

The online version of this article (doi:10.1186/s12866-015-0375-y) contains supplementary material, which is available to authorized users.

## Background

Rice blast caused by the heterothallic ascomycete *Magnaporthe oryzae*, is the most destructive disease of cultivated rice worldwide and can lead to severe losses of annual rice yield [1,2]. Under normal conditions, the fungus uses a highly specialized infection structure appressorium generated from a conidium for plant penetration [3,4]. After successful penetration, the invasive hyphae grow rapidly in the host cells and caused blast lesions. In 5 to 7 days, the pathogen produces numerous conidia from the lesions and initiates a new infection cycle.

Regulation of gene expression at the level of transcription controls many crucial biological processes. A number of different factors, including transcription factors, are essential for the process of transcription. Transcription factors can recognize DNA in a sequence-specific manner and modulate the frequency of initiation of transcription upon binding to specific sites in the promoter of target genes. The transcription factors can be activators, repressors, or both usually display a modular structure named the DNA-binding domain [5]. In *M. oryzae*, numerous transcription factors were identified and characterized to be important for proper regulation of infection related morphogenesis [6,7]. In our previous study, many transcription factors, including MoCrz1, MoAp1, MoAtf1, MoHac1, MoBzip10, MoSwi6 and MoMsn2 were reported to be involved in hyphal growth, asexual development, stress response, infectious growth and virulence by controlling the expression levels of a series of target genes [8-13]. In plants, Myb protein family comprises a large members of transcription factors [14]. The first identified *MYB* gene was the ‘oncogene’ v-Myb derived from the avian myeloblastosis virus [15]. Following v-Myb, a large and growing family of myb-related genes were discovered in a wide variety of eukaryotes including animals, plants, fungi and slime molds [16-18]. The Myb-related proteins contain a DNA-binding domain and generally function in the regulation of cell growth and differentiation, often by co-regulating gene expression along with DNA-binding proteins of other classes [19,20].

Myb proteins play important roles in controlling phenylpropanoid metabolism, cell shape, and hormonal responses during seed development and germination, and cellular proliferation in plants [21]. Additionally, two Myb proteins from fungi, Cdc5 and flbD were also reported to control cell shape [22,23]. In *Schizosaccharomyces pombe*, the Cdc5p is essential for G2/M progression and Cdc5 family members participate in a novel pathway to regulate G2/M progression [22,24]. In *Aspergillus nidulans*, *flbD* encodes a Myb-like DNA-binding protein and is required for early conidiophore development by activating a cascade of transcription factors for conidiophore production [22,23]. Here, we investigate the role of *MoMYB1* in growth and infection-related morphogenesis in *M. oryzae*. Deletion of *MoMYB1* resulted in a failure to develop conidiophores and conidia, and more tolerance to osmotic stressors. Furthermore, MoMyb1 plays a crucial role in cell wall integrity and tissue-specific infection of *M. oryzae*.

## Methods

### Fungal strains, cultures and transformation

The wild type strain Guy11, the mutants and the complemented transformants were cultured on complete medium (CM: 10 g D-glucose, 2 g peptone, 1 g yeast extract, 1 g casamino acids, 50 ml 20 × nitrate salts, 1 ml trace elements, 1 ml vitamin solution, 15 g agar, add distilled water to 1 L) [25] , straw decoction and corn powder medium (SDC: 100 g straw, 40 g corn powder, 15 g agar in 1 L distilled water), V8 juice agar medium (100 ml V8 juice, 900 ml ddH_2_O, 0.2 g CaCO_3_, 15 g agar) and oatmeal agar medium [26] at 28°C. Protoplast transformation was performed using hygromycin B (*HPH*) and bleomycin as selective marker for gene deletion and complementation assays as described [25]. Conidiation assays were performed as described [8].

### Deletion and complementation of *MoMYB1* in *M. oryzae*

The *MoMYB1* gene deletion mutants were generated using the standard one step gene replacement strategy as described [27]. The primer pairs FL4982/FL4983 and FL4984/FL4985 (Additional file 1: Table S1) were used to amplify the upstream and downstream flanking sequence, respectively. The hygromycin resistance gene cassette was prepared by primer pairs FL1111/FL1112 using *pfu* Taq DNA polymerase (TaKaRa) (Additional file 1: Table S1). The hygromycin resistant transformants were screened by genomic PCR, and further confirmed by RT-PCR and southern blot analysis. For complementation, the fragment containing the native promoter region and the entire open reading frame (ORF) of *MoMYB1* were amplified by primer FL4841/FL4842 (Additional file 1: Table S1) and inserted into the pYF11 vector with a bleomycin resistance gene [28], and then transformed into the Δ*Momyb1* mutant to obtain the complemented transformants.

### Pathogenicity assays

The two-week-old seedlings of susceptible rice cultivar CO-39 were used to perform the detached leaf infection assays. Mycelial plugs of the wild type Guy11, Δ*Momyb1* mutants and the complemented transformant were inoculated on the intact leaves and kept in a moist chamber at 28°C for 24 h in darkness, followed by a 12/12 hour light/dark cycle. Photographs were taken at 7 days after inoculation. Root infection assays were performed as described [29]. Lesion formation was examined at 9 days post-inoculation. The experiments were repeated three times. For infectious hyphal growth on rice roots, mycelia mats of Guy11 and ∆*Momyb1* expressing a GFP protein were cultured in liquid CM medium at 28°C for 2 days, then harvested and inoculated on the roots. After 48 h incubation under humid conditions at 28°C, the roots were observed under a fluorescence microscope.

### Osmoregulation and CFW assays

Osmoregulation and CFW assays were performed as described [30]. Briefly, strain blocks were placed onto the freshly prepared CM agar plates with NaCl (0.7 M), KCl (0.6 M), and sorbitol (1 M), respectively, and cultured in the dark at 28°C for 7 days. For CM medium containing cell wall perturbing agent Calcofluor White (CFW), the final concentrations were 200, 400, and 600 μg/ml of CFW, respectively. The sensitivity was evaluated by measuring the growth rate, and the experiments were repeated three times with three replicates each time.

### Cellular chitin content assay

Chitin (N-acetylglucosamine, GlcNAc) content was determined as described [31,32]. Mycelia were freeze-dried first. For each sample, 5 mg of dried biomass was resuspended in 1 ml 6% KOH and heated at 80°C for 90 min. Samples were centrifuged and pellets washed with PBS and resuspension. The pellets were finally resuspended in 0.5 ml of McIlvaine’s buffer with *Streptomyces plicatus* chitinase (Sigma, USA) and incubated for 16 hours at 37°C with gentle mixing. 100 ml sample was then combined with 100 ml of 0.27 M Mosadiumborate, heated for 10 min at 100°C, and 1 ml of freshly diluted (1:10) of Ehrlich’s reagent was added. After incubating at 37°C for 20 min, 1 ml of the sample was transferred to a 2.5 ml plastic cuvette (Greiner) and the absorbance at 585 nm was recorded. Standard curves were prepared with GlcNAc (Sigma, USA). The experiment was repeated three times.

### Protoplast release assay

The wild type Guy11 and the mutant strains were cultured in liquid CM media for 2 days and the mycelia were collected by centrifugation for 10 min at 5,000 rpm. The following lysis and protoplast release steps were performed as described previously [33,34]. The mycelia were washed twice and resuspended using 20% sucrose. Lysing enzyme from *Trichoderma harzianum* (Sigma-Aldrich, USA) was added to the suspension, with lysis stopped after 30, 60, and 90 min. Protoplast release were counted with a hemacytometer, and cell wall degradation was examined with the light microscope. The experiment was repeated three times.

### Nucleic acid manipulation

DNA extraction and DNA gel blot hybridization were performed using standard procedures as described [35]. Probe labeling, hybridization and detection were preformed with the DIG High Prime DNA Labeling and Detection Starter Kit (Roche Applied Science, Penzberg, Germany).

### Quantitative RT-PCR assay

Two-week-old rice seedlings were inoculated with a spore suspension of rice blast at 1 × 10^5^ spores/ml. The inoculated plants were placed in a chamber in the dark for 24 h at 25°C, and leaf tissues were collected at 8 h and 48 h after inoculation. Mycelia were grown in liquid complete medium for 48 h at 28°C, 150 rpm and harvested. Total RNA was extracted using the Invitrogen kit as described previously [12].

For quantitative RT-PCR (qRT-PCR), 5 mg of total RNA were reverse transcribed into first-strand cDNA using the oligo (dT) primer and M-MLV Reverse Transcriptase (Invitrogen). The qRT-PCR reactions were performed following previously established procedures [12]. To compare the relative abundance of target gene transcripts, the average threshold cycle (Ct) was normalized to that of *ACTIN* gene (MGG_03982) as described [36]. *P* < 0.01 is used in the statistical test. The primer pairs used in this section are listed in Additional file 1: Table S1.

### Bioinformatics

The full sequence of *MoMYB1* was downloaded from the *M. oryzae* online database (http://www.broadinstitute.org/annotation/genome/magnaporthe_grisea/MultiHome.html) [37]. Myb1 sequences from different organisms were obtained from GeneBank (http://www.ncbi.nlm.nih.gov/BLAST), using the BLAST algorithm [38]. Sequence alignments were performed using the Clustal_W 1.83 program [39].

## Results

### Identification of *M. oryzae MYB1*

The Myb family of proteins is a group of functionally diverse transcriptional activators that is characterized by a conserved DNA-binding domain of approximately 50 amino acids [14]. Here, we identified an *S. cerevisiae BAS1* homolog *MYB1* from the *M. oryzae* genome by a BLAST_P search. *MoMYB1* (MGG_06898.6) encoding a protein of 322 amino acids possesses two SANT (a putative DNA binding domain in the SWI-SNF and ADA complexes, the transcriptional co-repressor N-CoR and TFIIIB; InterPro: IPR001005) domains that are interrupted by one intron [40]. Alignment analysis showed that MoMyb1 shares similarities to the homologs from *S. cerevisiae*, *A. nidulans, Schizosaccharomyces pombe*, *Homo sapiens*, *Arabidopsis thaliana* and *Zea Mays* [22,23,41-45]*.* The amino acid sequence identity being 16%, 29%, 15%, 22%, 14%, and 21%, respectively. The alignment of repeat DNA-binding domains was shown in Additional file 2: Figure S1. Southern blot analysis revealed *MoMYB1* only had a unique copy in *M. oryzae* genome (Additional file 3: Figure S2B)

### MoMyb1 is highly expressed during conidia and plant colonization

**Figure 1:**
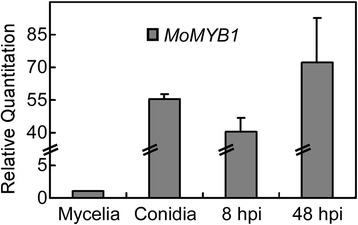
**Expression profiles of** ***MoMYB1*** **at different fungal developmental stages.** RNA was extracted from mycelia, conidia and infectious stages (8 and 48 hpi), respectively. *ACTIN* was used for normalization and values represent mean ± SD from two independent experiments with three replicates each. Asterisks were indicated significant differences at *P* < 0.01.

To gain insight into the functions of MoMyb1, we first examined the gene expression profile at hyphal, conidial and infectious stages of *M. oryzae* by qRT-PCR. In comparison to the hyphal stage (1.0 ± 0.1), the expression of *MoMYB1* was significantly increased in conidial and infection stages. The abundance of *MoMYB1* was increased by 55-fold (55.2 ± 2.6) in conidial stage and increased by 40-fold (40.4 ± 8.9, 8 hours post inoculation: hpi) and 71-fold (71.0 ± 23.5, 48 hpi) in the infectious stage (Figure 1).

### Targeted deletion of *MoMYB1* in *M. oryzae*

To evaluate the role of *MoMYB1* in growth and development of *M. oryzae*, deletion mutant were generated by replacing the *MoMYB1* gene with the hygromycin phosphotransferase resistance cassette (Additional file 3: Figure S2A). Hygromycin resistant transformants were first screened by genomic PCR and further confirmed by southern blot analysis and RT-PCR (Additional file 3: Figure S2B and S2C). Since all successful gene deletion mutants yield the same phenotypes, only one mutant was selected to analyze the biological phenotypes in this study. For complementation, a 2.8 kb fragment containing the native promoter and ORF of *MoMYB1* gene was cloned into pYF11 [28] and reintroduced into the Δ*Momyb1* mutant. The resulting transformants were confirmed by RT-PCR to obtain the complemented strain Δ*Momyb1/MoMYB1* (Additional file 3: Figure S2C).

### *MoMYB1* is involved in vegetative growth and is essential for conidiogenesis

**Figure 2:**
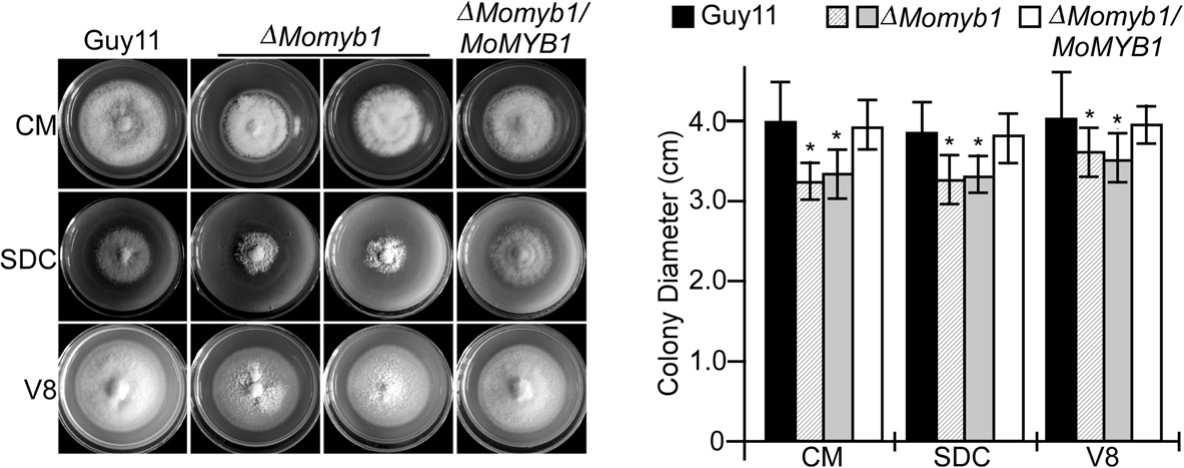
**Colony morphology and vegetative growth of Guy11,** Δ***Momyb1*** **and the complemented transformant (**Δ***Momyb1/MoMYB1*** **) on complete media (CM), straw decoction and corn (SDC) and 10% V8 juice agar media (V8).** Photographs were taken after 7-day incubation in the dark at 28°C. Asterisks indicated significant differences at *P* < 0.01. Values represent mean ± SD from three replicates each.

**Figure 3:**
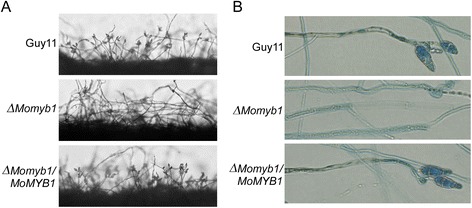
***MoMYB1*** **is required for conidiophore development. (A)** Conidia formation was observed under a light microscope 24 hours at room temperature after induction of conidiation on cover slips. The strains were first grown on SDC media for 7 days. **(B)** Aerial cultures stained with lactophenol cotton blue and observed under light microscope. Hyphae were stained blue, while conidiophores were in gray.

To determine whether *MoMYB1* was involved in growth and conidiation, the wild type Guy11, Δ*MoMyb1* mutants and the complemented transformant Δ*MoMyb1/MoMYB1* were inoculated on CM, SDC and V8 agar plates. Compared to Guy11 and Δ*MoMyb1/MoMYB1*, the Δ*MoMyb1* mutants showed significant reduced vegetative growth on these three media (Figure 2). The conidiation was also quantified on CM, V8, oatmeal (OM) and SDC media, the result revealed that the production of conidia in the Δ*Momyb1* mutants was completely abolished on these four media (Figure 3A). To find out the potential reasons of the conidiation defect, we further stained the aerial hyphe with lactophenol cotton blue as described [46], and no conidiophores was observed in the Δ*MoMyb1* mutants, while normal gray conidiophores were formed in the wild type Guy11 (Figure 3B).

### *MoMYB1* modulates the transcription of several conidiogenesis-related genes

**Figure 4:**
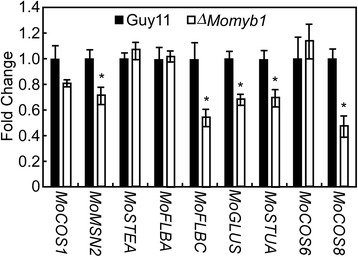
***MoMYB1*** **regulates the transcription of conidiation-related homologous genes.** RNA was extracted from mycelia cultured in liquid CM medium at 28°C for 2 days. *ACTIN* was used for normalization, and the values were calculated by 2^-ddCT^ methods with quantitative RT-PCR data. Values represent mean ± SD from two independent experiments with three replicates each. Asterisks were indicated significant differences at *P* < 0.01.

Because MoMyb1 is a putative Myb transcription factor, which is required for an early stage of conidiation, we speculate that MoMyb1 acts as a transcription factor that regulates the expression of other conidiation-related genes. To test this hypothesis, the expression of several conidiation-related genes or their orthologs was analyzed [11]. The results revealed that *MoMSN2*, a homologue of *ScMSN2* of *S. cerevisiae*, and *MoFLBC,* homologous to *FLBC* of *A. nidulans*, and glutamine synthetase (*MoGLUS*), homologous to *FLUG* of *A. nidulans*, and *MoSTUA*, homologous to *STUA* of *A. nidulans*, and *MoCON8*, homologous to *CON8* of *N. crassa,* was significantly down-regulated in the Δ*MoMyb1* mutant (Figure 4).

### *MoMYB1* plays an essential role in virulence on rice roots but not on leaves

**Figure 5:**
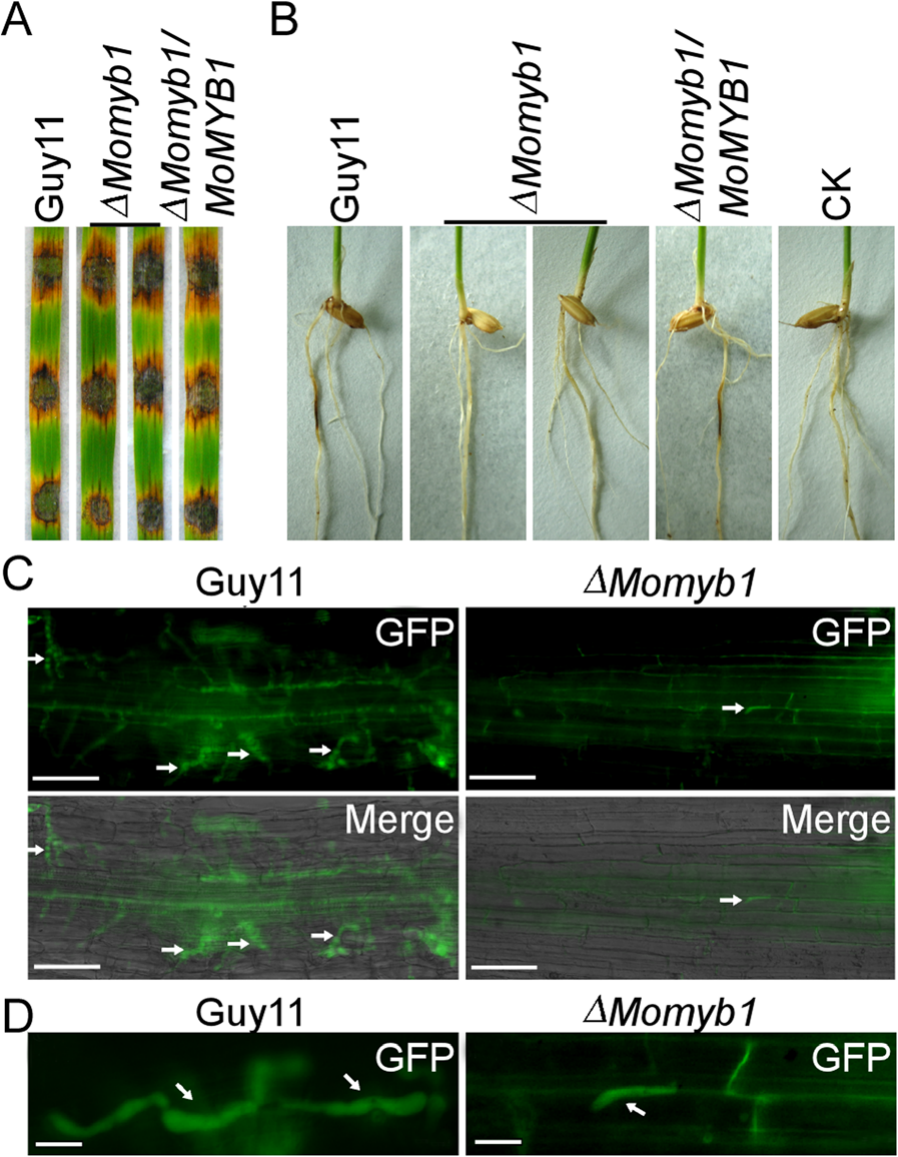
**Effect of** ***MoMYB1*** **deletion on pathogenicity. (A)** Detached rice leaf assay. Intact rice leaves were inoculated by mycelium plugs from wild type Guy11, Δ*Momyb1* mutant and complemented transformant Δ*Momyb1/MoMYB1*. Photographs were taken at 7 days after inoculation. **(B)** Rice root infection assay. Lesions were examined at 9 days post-inoculation. CK: inoculated with agar plugs without hyphae. **(C)** Observation of the invasive hyphal growth inside the rice root inoculated with the Guy11 and Δ*Momyb1* strain expression a GFP protein, respectively. White arrows point to invasive hyphae. Bar = 50 μm. **(D)** Close view the invasive hyphae in plant cells. White arrows point to invasive hyphae. Bar = 10 μm.

Since Δ*Momyb1* mutant have a conidiation defect, the mycelial plugs of the wild type Guy11, Δ*Momyb1* mutant and the complemented transformant were inoculated on the detached rice leaves to test the pathogenic abilities. After 7 days incubation, the Δ*Momyb1* mutants caused large and extended lesions similar to that of the wild type Guy11 and the complemented transformant (Figure 5A). We further examined the pathogenicity of the Δ*Momyb1* mutant on rice roots. Unlike to the results on rice leaves, the Δ*Momyb1* mutant caused no virulence on roots 9 days after inoculation. In contrast, the wild type Guy11 and the complemented transformant caused typical rice blast lesions under the same conditions (Figure 5B). These results suggested that MoMyb1 played an important role in tissue-specific infection of *M. oryzae*. To confirm this result, we observed the infectious hyphal growth in the rice root by transforming a green fluorescence protein (GFP) into wild type Guy11 and Δ*Momyb1* mutant, respectively. The results showed that the wild type could penetrate through the root epidermis and formed branching invasive hyphae at 48 h, while successful penetration and development of invasive hyphae were rarely observed in the Δ*Momyb1* mutant (Figure 5C and D).

### Deletion of *MoMYB1* results in more insensitive to salt and osmotic stresses

**Figure 6:**
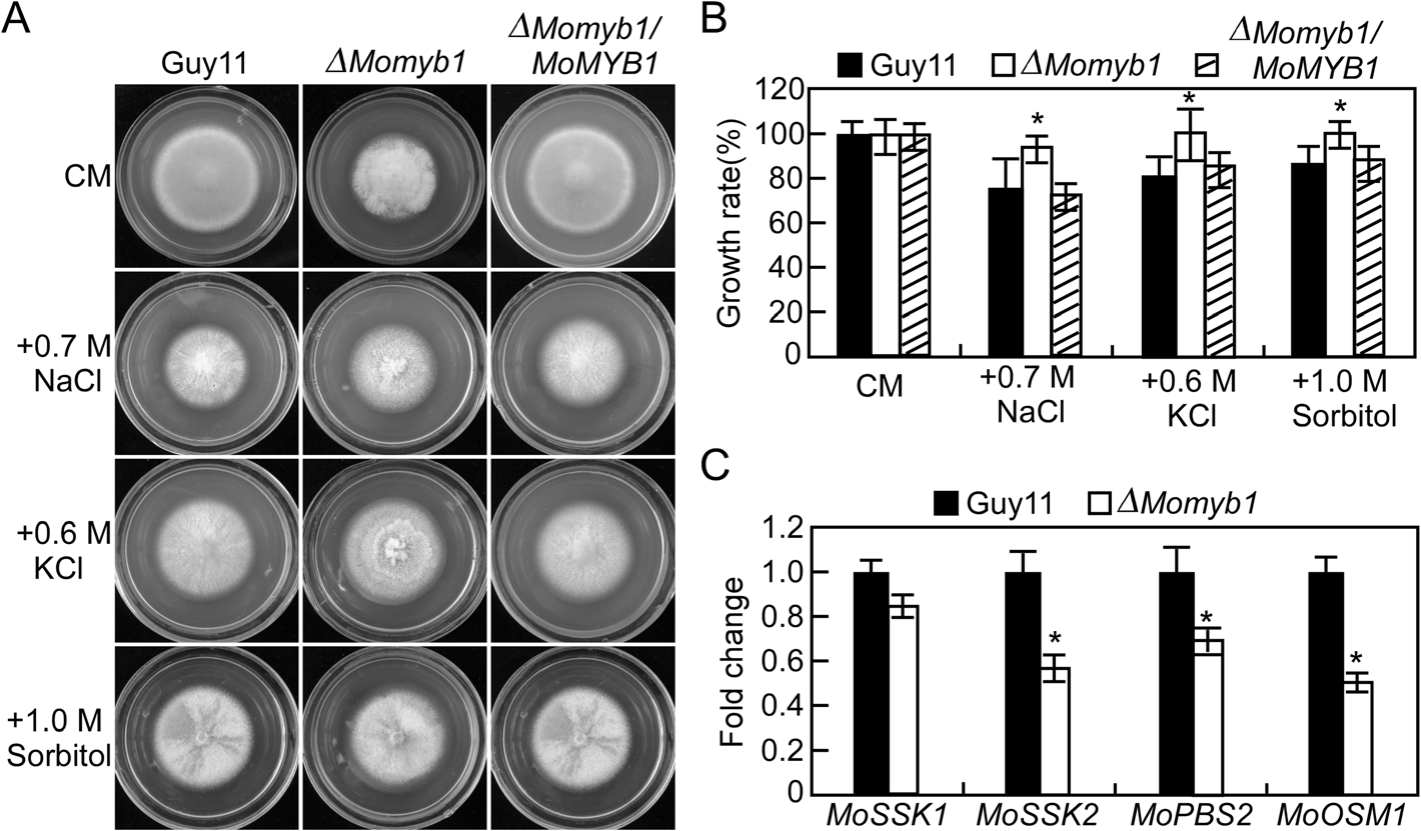
Δ***Momyb1*** **mutants are more insensitive to osmotic stresses. (A)** Wild type Guy11, Δ*Momyb1* mutants and complemented transformant were incubated on CM plates containing various concentrations of NaCl, KCl or sorbitol at 28°C for 7 days. **(B)** The growth rate was determined 7 days after incubation at 28°C by plotting the percentage of colonies in the presence of various concentrations of NaCl, KCl or sorbitol against regular CM. **(C)** qRT-PCR analysis the transcription of four components of the Hog1 pathway in *M. oryzae*. Asterisks were indicated significant differences at *P* < 0.01.

To address the role of *MoMYB1* in environmental adaptation, the wild-type Guy11, Δ*Momyb1* mutant and the complemented transformant were inoculated on the CM agar plates containing the salt (0.7 M NaCl, 0.6 M KCl) and osmotic (1 M sorbitol) stresses, respectively. Compared with the wild-type and the complemented transformant, the Δ*Momyb1* mutant showed less sensitivity to NaCl, KCl and sorbitol (Figure 6A). The growth rate of the Δ*Momyb1* mutant was much higher than that of wild type and the complemented transformant (Figure 6B). This result suggested that *MoMYB1* has a crucial role in response to salt and osmotic stresses. Since Hog1 pathway was the most important signal pathway to responsible for stress response in *M. oryzae* [6], we examined the expression of four major components of the pathway, including *MoSSK1*, *MoSSK2*, *MoPBS2* and *MoOSM1* (*MoHOG1*). The results revealed that, besides *MoSSK1*, the expression levels of *MoSSK2*, *MoPBS2* and *MoOSM1* were significantly decreased in the Δ*Momyb1* mutant compared to the wild type Guy11 (Figure 6C).

### MoMyb1 is involved in cell wall integrity

**Figure 7:**
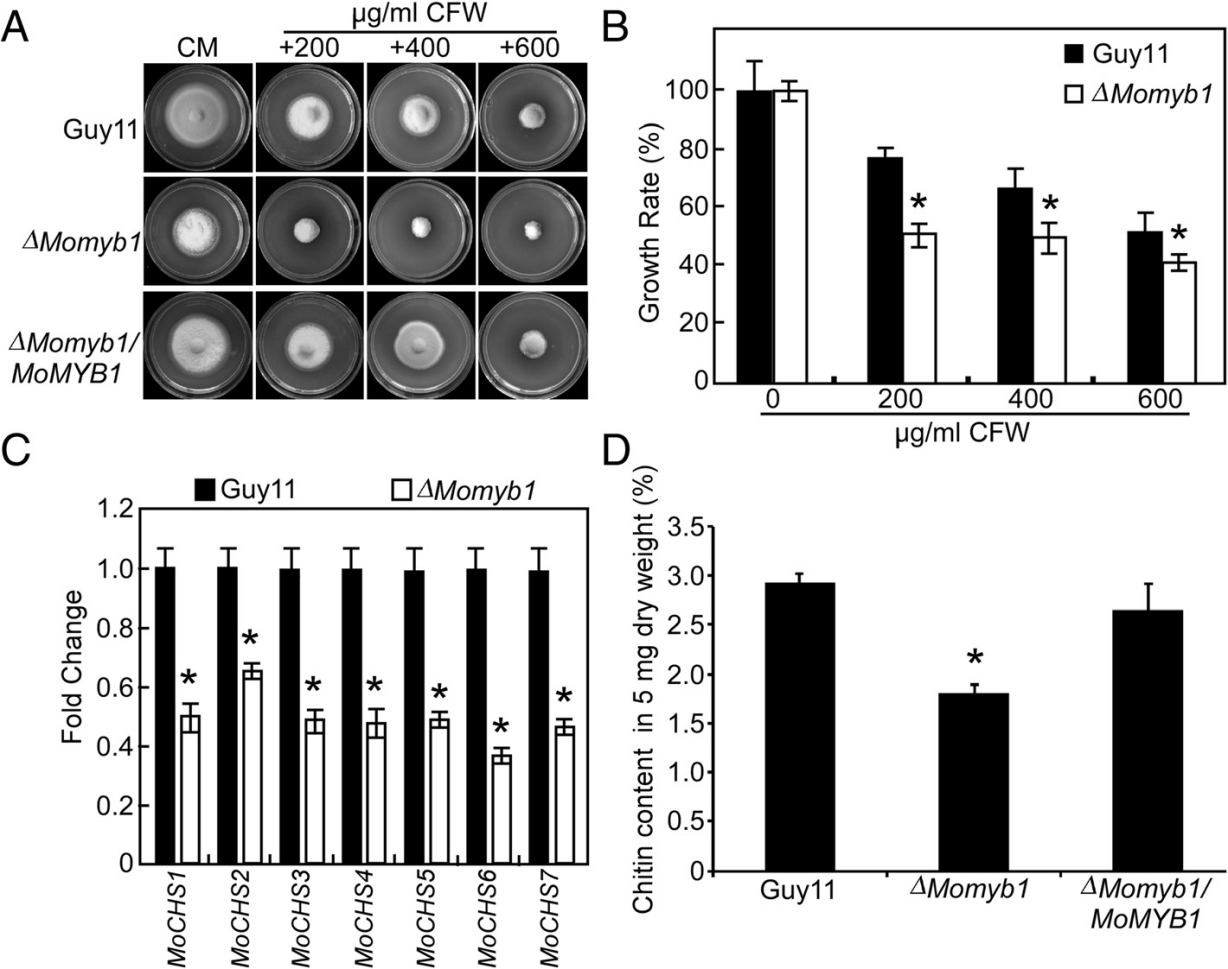
**MoMyb1 has a role in cell wall integrity. (A)** Wild type Guy11, Δ*Momyb1* mutant and the complemented transformant were incubated on CM plate containing different concentrations of CFW at 28°C for 7 days. **(B)** The growth rate was determined 7 days after incubation at 28°C by plotting the percentage of colonies in the presence of various concentrations of CFW against regular CM. **(C)** The expression levels of seven chitin synthases encoding genes in the ∆*Momyb1* mutant. **(D)** GlcNa determination shows significantly decreased chitin contents in the ∆*Momyb1* mutant. Data comprise three independent experiments with triple replications each time that yielded similar results. Asterisks were indicated significant differences at *P* < 0.01.

**Figure 8:**
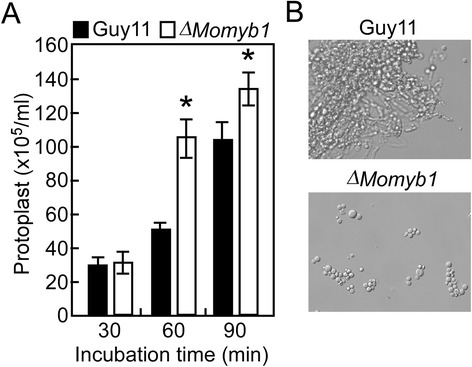
**Protoplast release of the wild type Guy11 and ∆** ***Momyb1*** **mutant. (A)** Protoplast production of Guy11 and ∆*Momyb1* mutant treated by cell wall degrading enzyme. Asterisks indicate a significant difference between the mutant and wild-type strain at *P* < 0.01. **(B)** Light microscopic examination of protoplast release after treatment for 60 min and photographed.

To determine whether *MoMYB1* has a role in maintenance cell wall integrity, we test mycelial growth on CFW which inhibit fungal cell wall assembly by binding chitin. The results showed that the growth rate of the Δ*Momyb1* mutant on CFW media was significantly decreased, which reduced to 66.7%, 70.8% and 85.4% of the wild type Guy11 under 200, 400, and 600 μg/ml CFW, respectively, while the complemented transformant Δ*Momyb1/MoMYB1* can fully restore the defects (Figure 7A and B). Chitin is a major component of fungal cell wall and is synthesized by chitin synthases and *M. oryzae* contains seven chitin synthases [47]. Therefore, we examined the transcription levels of these chitin synthase genes by qRT-PCR analysis. The results revealed that the expression of all chitin synthase genes was significantly decreased in the Δ*Momyb1* mutant (Figure 7C). We also examined the chitin content of the mutant and found the chitin content was remarkably reduced in the Δ*Momyb1* mutant compared to the wild type (Figure 7D), indicating *MoMYB1* has a role in cell wall assembly. To further confirm this conclusion, the mycelia of wild type Guy11 and the Δ*Momyb1* mutant were treated with cell wall degrading enzyme. The results showed that the Δ*Momyb1* mutant was more sensitive to the enzyme treatment and released more protoplast after incubation for 60 and 90 min compared to the wild type Guy11 (Figure 8A). When observed at 60 min, the Δ*Momyb1* mutant displayed a greater number of protoplasts and no mycelia fragments were observed. In contrast, the wild type showed much less protoplasts and many mycelial fragments were found under the same condition (Figure 8B).

## Discussion

In the present study, we characterized a Myb family protein, MoMyb1 in *M. oryzae* and primarily focused on its external phenotypes associated with pathogenesis. Gene-targeted replacement revealed that the loss of *MoMYB1* led to a plethora of developmental defects in vegetative growth, conidiation, stress response, cell wall integrity and virulence.

Conidiogenesis and appressorium development are key steps in the rice blast disease cycle. The fungus has evolved regulatory networks to ensure the correct timing and spatial pattern of these development events [7]. The expression profile of *MoMYB1* at hyphal, conidial and infectious stages of *M. oryzae* suggested that MoMyb1 might have a crucial role in conidial development and plant infection. Deletion of *MoMYB1* in *M. oryzae* affected different developmental stages, including hyphal growth, cell wall assembly and penetration. In addition, the Δ*Momyb1* mutants displayed no conidia and conidiophores, suggesting that deletion of *MoMYB1* in *M. oryzae* completely abolished conidiophore development and thus affected conidium production. Therefore, we conclude that MoMyb1 is a key regulator of *M. oryzae* for conidium and conidiophore development*.* qRT-PCR analysis revealed the expression level of five conidiogenesis-related genes were significantly decreased in the ∆*Momyb1* mutant, indicating that MoMyb1 probably functions as a key upstream transcription factor in the conidiogenesis signalling pathway to regulate the expression of genes which involved in conidiophore and conidium development. However, whether these conidiogenesis-related genes directly regulated by MoMyb1 need further studies. One surprise result was that the ∆*Momyb1* mutant caused rice blast on rice roots, but not on leaves. Since the infectious mechanism of *M. oryzae* on rice roots has been well clarified [48], we conclude that the pathogenic difference in ∆*Momyb1* on the two organs is a result of tissue-specific infectious-related development mechanisms.

Cell fate specification is a process of fundamental importance during development. In maize, GL1 encodes a protein containing a Myb DNA-binding domain and is expressed most highly very early during trichome development and is a central regulator of the trichome cell fate decision [49]. Two Myb proteins from fungi, the *CDC5* gene product from *S. pombe* [22] and the *FLBD* gene product from *A. nidulans* [23] can also control aspects of cell shape, indicating Myb proteins are related to cell wall integrity. Our results show that MoMyb1 is involved in the response to multiple stresses via regulating different signalling pathways including Hog1 pathway, which contributes to osmoregulation. The insensitive or hypersensitive of ∆*Momyb1* mutant to variety of stresses may also indicate that *MoMYB1* is involved in cell wall integrity.

Chitin is an integral part of the fungal cell wall and its synthesis depends on the activity of chitin synthase enzymes. In the present study, the ∆*Momyb1* deletion mutants showed high sensitivity to the cell wall stressor CFW. In *M. oryzae*, seven chitin synthases encoding genes were characterized to involve in the development and pathogenicity by affected chitin content [47]. In other phytopathogenic fungi, chitin synthases also play crucial roles in proper regulation of infection-related morphogenesis [50-54]. In the ∆*Momyb1* mutant, the transcriptional levels of seven chitin synthases were down-regulated and the chitin content was also decreased in the mutant, suggesting that MoMyb1 may be a multi-stress regulator of *M. oryzae* that contributes to the loss of pathogenicity. MoMyb1 might directly or indirectly regulate chitin synthesis, thus may disturb the responses of cell wall signalling pathways to different stressors.

## Conclusion

This study demonstrated that MoMyb1 functions as a key regulator that important for vegetative growth, conidiogenesis, stress response and pathogenicity in *M. oryzae*. MoMyb1 is also involved in the maintenance of cell wall integrity that are crucial for the growth and development of the fungus.

## Additional files

Additional file 1: Table S1. Primers used in this study. Additional file 2: Figure S1. Alignment of gene encoding repeats domain among *M. oryzae* and other organisms. The predicted amino-terminal sequence of MoMyb1 is compared with that of various Myb-like proteins: *Aspergillus nidulans* FlbD [23]; *Homo sapiens* c-Myb [41,42]; *Arabidopsis thaliana* Atr1 [43]; *Zea Mays* Cl [44]; *Schizosaccharomyces pombe* Cdc5 [22]; *S. cerevisiae* Bas1 [45]. The two repeating motifs of ~50 amino acids are grouped, and the amino acids identical in five or more are showed in grey bars. Additional file 3: Figure S2. Generation of *MoMYB1* deletion mutant. (A) Restriction map of the *MoMYB1* genomic region. Disruption constructs containing the homologous sequences flanking the *HPH* cassette to replace *MoMYB1*. (B) Southern blot analysis of the wild type (lane 1, 2, 3 and 6) and ∆*Momyb1* mutant (lane 4, 5, 7 and 8) using *MoMYB1* and *HPH* specific probes, respectively. Genomic DNA was digested with *EcoR*I, *Sal*I and *Nde*I*,* respectively. (C) RT-PCR analysis of *MoMYB1* in wild type, ∆*Momyb1* mutants and the complemented transformant. The transcript was lost in the ∆*Momyb1* mutants.

## Abbreviations

qRT-PCR: Quantitative reverse transcription polymerase chain reaction
CFW: Calcofluor white
DNA: Desoxyribonucleic acid
ORF: Open reading frame
BLAST: Basic local alignment search tool
SANT: a putative DNA binding domain in the SWI-SNF and ADA complexes, the transcriptional co-repressor N-CoR and TFIIIB
