# Correction to: MoMyb1 is required for asexual development and tissue-specific infection in the rice blast fungus Magnaporthe oryzae

**DOI:** 10.1186/s12866-018-1263-z

**Published:** 2018-10-03

**Authors:** Yanhan Dong, Qian Zhao, Xinyu Liu, Xiaofang Zhang, Zhongqiang Qi, Haifeng Zhang, Xiaobo Zheng, Zhengguang Zhang

**Affiliations:** 1 Department of Plant Pathology, College of Plant Protection, Nanjing Agricultural University, Nanjing, 210095 China; 2 Key Laboratory of Integrated Management of Crop Diseases and Pests, Ministry of Education, Nanjing, 210095 China

## Correction

Following the publication of this article [1], the authors noticed that they mistakenly introduced duplicate images in Fig. 6a during the preparation of figures. They apologize for any confusion that brought to the readers and have corrected the figure. This correction does not change any statement or conclusion drawn from the data.

**Fig. 6:**
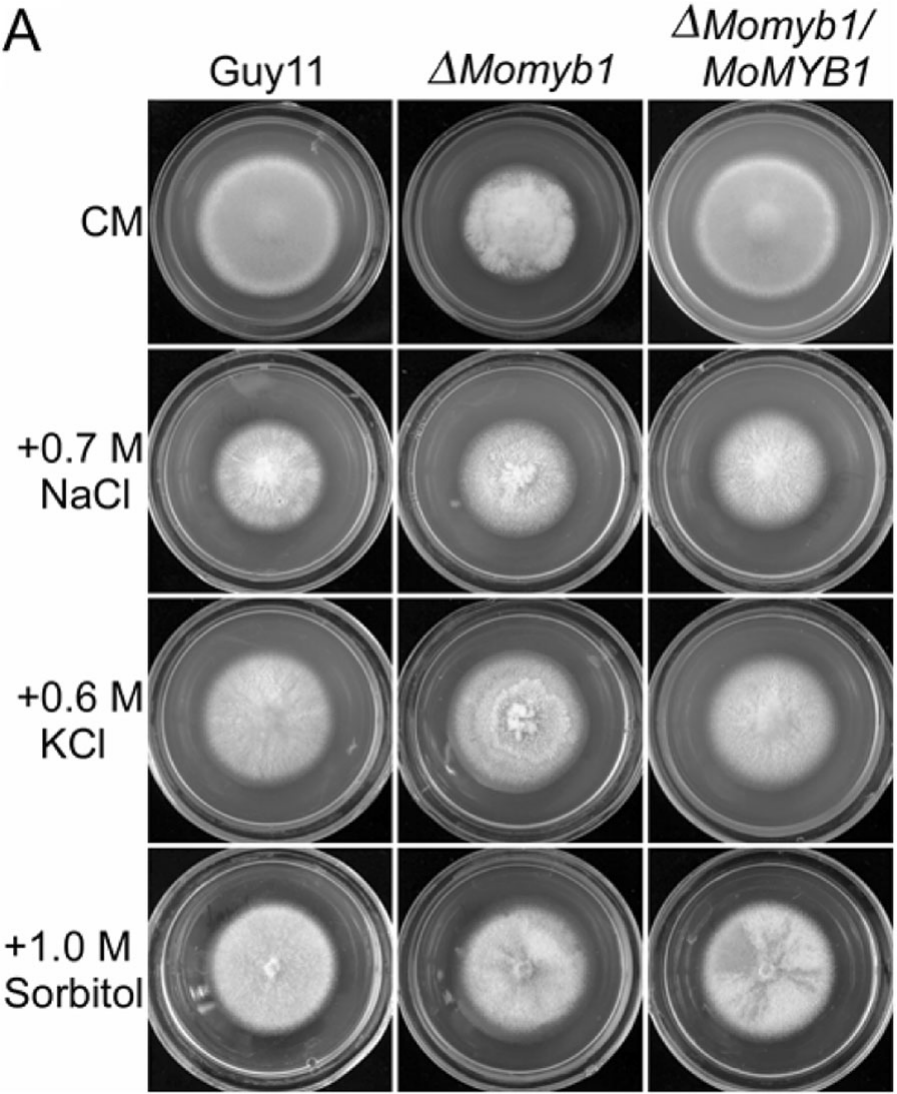
Δ*Momyb1* mutants are more insensitive to osmotic stresses. **a** Wild type Guy11, Δ*Momyb1* mutants and complemented transformant were incubated on CM plates containing various concentrations of NaCl, KCl or sorbitol at 28°C for 7 days. **b** The growth rate was determined 7 days after incubation at 28°C by plotting the percentage of colonies in the presence of various concentrations of NaCl, KCl or sorbitol against regular CM. **c** qRT-PCR analysis the transcription of four components of the Hog1 pathway in M. *oryzae*. Asterisks were indicated significant differences at *P* < 0.01

The correct version of Fig. 6a has been included in this correction.
