# Supplementary material for: MoMyb1 is required for asexual development and tissue-specific infection in the rice blast fungus Magnaporthe oryzae
Source: BMC Microbiol. 2015 Feb 19;15:37. doi: 10.1186/s12866-015-0375-y (PMC4336695; doi:10.1186/s12866-015-0375-y)
Supplement: Additional file 1: Table S1. — Primers used in this study. [file 12866_2015_375_MOESM1_ESM.docx]

Table S1. Primers used in this study.

| Primer | Sequence(5’-3’) | Application |
| --- | --- | --- |
| FL4982 | CTTGTTTCTCCCGCTGCATG | amplify *MYB1* 5’ flank sequence |
| FL4983 | TGCTTGAAATAAACAAACATGATATCGTTGAAATCCTGATGATAAT | amplify *MYB1* 5’ flank sequence |
| FL4984 | ATTATCATCAGGATTTCAACGATATCATGTTTGTTTATTTCAAGCA | amplify *MYB1* 3’ flank sequence |
| FL4985 | AATGTGGTAGAGGATCAGAT | amplify *MYB1* 3’ flank sequence |
| FL4841 | ACTCACTATAGGGCGAATTGGGTACTCAAATTGGTT GACCTAACCACCAGTCTATCTAG | *MYB1*-GFP fusion for complementation |
| FL4842 | CACCACCCCGGTGAACAGCTCCTCGCCCTTGCTCAC GTTCATGATGGAGGCGATCGAG | *MYB1*-GFP fusion for complementation |
| FL4747 | ACCAGTTCAACAACCCTTCG | RT-PCR for *MYB1* |
| FL4748 | ACTGCTTGGAGATGGGTGAC | RT-PCR for *MYB1* |
| FL1111 | GGAGGTCAACACATCAATG | amplify *HPH* cassette |
| FL1112 | CTCTATTCCTTTGCCCTCG | amplify *HPH* cassette |
| COS1-QF | CCCTCAGCCCACATACAACT | quantitative RT-PCR analysis |
| COS1-QR | AGCCTTCGCTCGATACTGAA | quantitative RT-PCR analysis |
| STEA-QF | CAGTCCACTTCCCCTGTCAT | quantitative RT-PCR analysis |
| STEA-QR | GGGACTCCGTTCTGGTTGTA | quantitative RT-PCR analysis |
| FLBA-QF | AACGGACAAGTTGGTCAAGG | quantitative RT-PCR analysis |
| FLBA-QR | GCCATCTTTACTCCGGTCAA | quantitative RT-PCR analysis |
| FLBC-QF | CTAGCCAGGGTATGCTTTCG | quantitative RT-PCR analysis |
| FLBC-QR | TGCTAGCCAATGACTCAACG | quantitative RT-PCR analysis |
| MSN2-QF | GTGCCTAGCAACGAGGAGAC | quantitative RT-PCR analysis |
| MSN2-QR | CTCAAAGGGCTTGTCCTGAG | quantitative RT-PCR analysis |
| GLUS-QF | TGCGAAAAGTTCAACTGCAA | quantitative RT-PCR analysis |
| GLUS-QR | CTTAGGCTTTGCCATGAAGC | quantitative RT-PCR analysis |
| STUA-QF | CAACATGGGCAGCTCTGATA | quantitative RT-PCR analysis |
| STUA-QR | CCTGCATGCTTTGTAGCGTA | quantitative RT-PCR analysis |
| CON6-QF | CACAAGGCCAACCTCAA | quantitative RT-PCR analysis |
| CON6-QR | TCTCCATCTCCTCGAGAC | quantitative RT-PCR analysis |
| CON8-QF | CGACCTCTTCCTCTTCTTCG | quantitative RT-PCR analysis |
| CON8-QR | TTCCACATCTTGCCAAACAG | quantitative RT-PCR analysis |
| ACTIN-QF | CCATGTACCCTGGTCTTTCG | quantitative RT-PCR analysis |
| ACTIN-QR | TTCGAGATCCACATCTGCTG | quantitative RT-PCR analysis |
| CHS1-QF | TGCTGCTCATGTCCACCTAC | quantitative RT-PCR analysis |
| CHS1-QR | TCCTCTTGAGGCTTGTCGAT | quantitative RT-PCR analysis |
| CHS2-QF | CTTGTTGCAAAGCGAGATGA | quantitative RT-PCR analysis |
| CHS2-QR | CCTGGAGAAGCTGGTAGACG | quantitative RT-PCR analysis |
| CHS3-QF | CGACCAGCTTCAACTTCACA | quantitative RT-PCR analysis |
| CHS3-QR | GGAGTCTGAGCTTCGTTTGG | quantitative RT-PCR analysis |
| CHS7-QF | AAACTCGAGGGACATGTTGG | quantitative RT-PCR analysis |
| CHS7-QR | CCTCCTGAACGCAGAGAAAC | quantitative RT-PCR analysis |
| CHS4-QF | TCCTGATGTCGTTCTTGCAG | quantitative RT-PCR analysis |
| CHS4-QR | GATCTCAGGGTCCTTCACCA | quantitative RT-PCR analysis |
| CHS6-QF | TATGCGCTACGATGACAAGC | quantitative RT-PCR analysis |
| CHS6-QR | CGAGTAAACCTTGCCCATGT | quantitative RT-PCR analysis |
| CHS5-QF | CACCTGCGTTTACCTTGGAT | quantitative RT-PCR analysis |
| CHS5-QR | TACCCCACGAGAAGTTGTCC | quantitative RT-PCR analysis |
| HOG1-QF | CGTGGCCTGAAATACGTCCACT | quantitative RT-PCR analysis |
| HOG1-QR | CCAGATATCTACCTCGACGTCG | quantitative RT-PCR analysis |
| SSK2-QF | GTACACCAACGGTGGAGCCACT | quantitative RT-PCR analysis |
| SSK2-QR | CCATAACCGTTGCGCATTCTAG | quantitative RT-PCR analysis |
| SSK1-QF | ACCTGCTCCGCATGTAGCTACT | quantitative RT-PCR analysis |
| SSK1-QR | ATGGAACCCTCCTTGACGCCA | quantitative RT-PCR analysis |
| PBS2-QF | TTCAGCATCTCGCTCGACGAGA | quantitative RT-PCR analysis |
| PBS2-QR | AGGATGGTCGTAAACTTGGCCT | quantitative RT-PCR analysis |
